# Analysis of miRNA and mRNA Expression Profiles Highlights Alterations in Ionizing Radiation Response of Human Lymphocytes under Modeled Microgravity

**DOI:** 10.1371/journal.pone.0031293

**Published:** 2012-02-09

**Authors:** Cristina Girardi, Cristiano De Pittà, Silvia Casara, Gabriele Sales, Gerolamo Lanfranchi, Lucia Celotti, Maddalena Mognato

**Affiliations:** 1 Dipartimento di Biologia, Università degli Studi di Padova, Padova, Italy; 2 Laboratori Nazionali di Legnaro, INFN, Padova, Italy; Louisiana State University and A & M College, United States of America

## Abstract

**Background:**

Ionizing radiation (IR) can be extremely harmful for human cells since an improper DNA-damage response (DDR) to IR can contribute to carcinogenesis initiation. Perturbations in DDR pathway can originate from alteration in the functionality of the microRNA-mediated gene regulation, being microRNAs (miRNAs) small noncoding RNA that act as post-transcriptional regulators of gene expression. In this study we gained insight into the role of miRNAs in the regulation of DDR to IR under microgravity, a condition of weightlessness experienced by astronauts during space missions, which could have a synergistic action on cells, increasing the risk of radiation exposure.

**Methodology/Principal Findings:**

We analyzed miRNA expression profile of human peripheral blood lymphocytes (PBL) incubated for 4 and 24 h in normal gravity (1 g) and in modeled microgravity (MMG) during the repair time after irradiation with 0.2 and 2Gy of γ-rays. Our results show that MMG alters miRNA expression signature of irradiated PBL by decreasing the number of radio-responsive miRNAs. Moreover, let-7i*, miR-7, miR-7-1*, miR-27a, miR-144, miR-200a, miR-598, miR-650 are deregulated by the combined action of radiation and MMG. Integrated analyses of miRNA and mRNA expression profiles, carried out on PBL of the same donors, identified significant miRNA-mRNA anti-correlations of DDR pathway. Gene Ontology analysis report**s** that the biological category of “Response to DNA damage” is enriched when PBL are incubated in 1 g but not in MMG. Moreover, some anti-correlated genes of p53-pathway show a different expression level between 1 g and MMG. Functional validation assays using luciferase reporter constructs confirmed miRNA-mRNA interactions derived from target prediction analyses.

**Conclusions/Significance:**

On the whole, by integrating the transcriptome and microRNome, we provide evidence that modeled microgravity can affects the DNA-damage response to IR in human PBL.

## Introduction

Eukaryotic cells have evolved efficient DNA-damage response to genotoxic agents in order to eliminate any detrimental effect of DNA lesions. Ionizing radiation (IR) in out-of Earth represents an environmental mutagen to which humans are daily exposed on Earth. Crewmembers of space mission are even more exposed to IR because the cosmic radiation field is rather different from that experienced on Earth, and fragmentation with spacecraft shielding modifies the radiation quality spectrum, hence modifying the biological effectiveness of IR in a manner that is still undetermined. The cell response to the space environment, which is characterized by a condition of weightlessness (i.e. microgravity, 10^−4^–10^−6^ g), includes immune cell function suppression [1], [2], skeletal muscle atrophy [3]–[5], cardiovascular disorders [6], loss of bone [7], [8], changes of gene expression [9], [10], increase in chromosomal aberrations and apoptosis [11], [12]. Studies carried out with systems simulating on Earth some aspects of microgravity, such clinostats and Rotating Wall Vessel bioreactors, reported similar results, indicating that the experiments performed with modeled microgravity can be used as surrogate of space conditions [13]–[20]. Despite the abundance of data about the biological effects of space and simulated microgravity, it is still unclear whether microgravity can affects the DNA-damage response (DDR) to IR. DDR is a complex pathway addressed to maintain genome integrity through the activation of proteins involved in sensing, signaling, and transducing the DNA damage signal to effector proteins of cell cycle progression/arrest, DNA repair and apoptosis [21]. While several studies reported additive/synergistic interactions of radiation and microgravity in different biological systems [22]–[26], other studies did not report such interactions [27]–[29]. In particular, the repair of radiation-induced DNA damage seems to be unaffected by microgravity in bacteria and human fibroblasts [30], [31] and in yeast [32]. On the contrary, in our previous work we detected a significant delay in the rejoining of DNA double-strand breaks induced by IR in human peripheral blood lymphocytes incubated in microgravity conditions [33]. Recent findings show the tendency of radioadaptation to DNA damage when space flown cells recovered on Earth are exposed to subsequent irradiation [34]. By considering the complexity of DDR and the controversial impact of reduced gravity on radio-sensitivity, we expected that the analysis of microRNA (miRNA) profiles could contribute to increase our knowledge on the features of space environment.

MiRNAs are endogenous small noncoding RNAs (18–24 nt), acting as post-transcriptional modulators of gene expression, by pairing to target mRNAs and leading to decreased translational efficiency and/or decreased mRNA levels [35]. A single miRNA can influence the expression of up to thousand genes, thus the function of a miRNA is ultimately defined by the genes it targets. Besides a physiological role of miRNAs in a variety of important biological processes including differentiation, apoptosis [36], fat metabolism [37], viral infection [38] and pathological processes, such as tumorigenesis [39]–[43], the miRNA-mediated gene regulation operates also in response to cellular stress. Ionizing radiation induces changes in miRNA expression both *in vitro* and *in vivo*, according to cell type, radiation dose and post-irradiation time [44]–[49]. Several studies suggested that miRNA expression is regulated in the DDR at the transcriptional level, in a p53-dependent manner [50], and through modulation of miRNAs processing and maturation steps [51]. Whilst miRNA-mediated DDR has been studied after ionizing radiation, UV radiation and hypoxic stress [52], [53], the response to IR combined with microgravity has not been studied yet, and should give important information about the risk of the exposure to space environment.

In our previous studies carried out with peripheral blood lymphocytes (PBL) incubated in modeled microgravity (MMG) during the repair time after IR, we reported significant decrease of cell survival, delay of double strand break repair, increase of mutant frequency and apoptosis [33], [54]. In the present study we analyzed miRNA expression profile of human PBL irradiated *in vitro* with 0.2 and 2Gy of γ-rays and incubated for a short (4 h) and medium-long (24 h) period in MMG and in parallel ground conditions (1 g). The results obtained from this study show differences in miRNA expression profile as a function of the dose and the time after irradiation in both gravity conditions. Interestingly, under MMG many miRNAs were not responsive to radiation compared with 1 g-condition. Analysis of mRNA expression profiles and further miRNA-mRNA anti-correlation analyses allowed the identification of DDR genes differently modulated in the two gravity conditions.

## Methods

### Ethics Statement

Human peripheral blood lymphocytes (PBL) were obtained from freshly collected “buffy coats” of healthy donors at the Blood Centre of the City Hospital of Padova (Italy). This study obtained ethics approval from the Transfusion Medicine (TM) ethics committee of Blood Centre of the City Hospital of Padova. The informed consent from donors was not required by the TM/ethics committee because PBL samples were analyzed anonymously.

### Cells, irradiation and microgravity simulation

**Figure 1:**
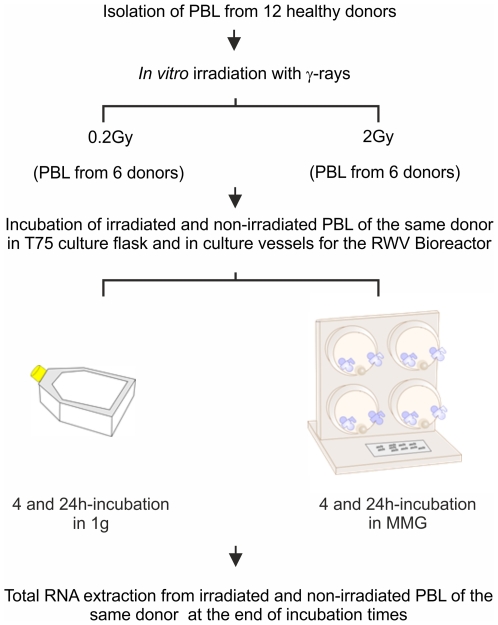
Experimental procedure of irradiation and microgravity simulation.

PBL were isolated by separation on Biocoll (Biochrom KG, Seromed) density gradient from freshly collected buffy coats from 12 healthy donors. After overnight incubation, PBL, consisting of peripheral mononuclear cells depleted of monocytes, were irradiated with γ-rays (0.2 and 2Gy) at the Department of Oncological and Surgical Sciences of Padova's University with a ^137^Cs source (dose-rate: 2.8Gy/min). PBL from six donors (named D, E, F, I, L, M) were irradiated with 0.2Gy whereas PBL from other six donors (named A, B, C, G, H, P) were irradiated with 2Gy, for a total of 12 independent experiments. For each experiment, irradiated and non-irradiated PBL of the same donor were incubated in 1 g and MMG conditions for 4 and 24 h. MMG was simulated by the Rotating Wall Vessel (RWV) bioreactor (Synthecon, Cellon), as previously described [14], [20]. PBL incubated in 1 g, irradiated and non, were kept in 75 cm^2^ flasks at the same density (Figure 1). In all experiments were used unstimulated quiescent (G_0_) PBL.

### Total RNA isolation

At the end of incubation time (4 and 24 h) in 1 g and MMG, total RNA was isolated from 10^7^ irradiated and non-irradiated PBL, by using Trizol® Reagent (Invitrogen, CA), according to the manufacturer's protocol. Total RNA quantification was performed using the ND-1000 spectrophotometer (Nanodrop, Wilmington, DE); RNA integrity and the content of miRNAs were assessed by capillary electrophoresis using the Agilent Bioanalyzer 2100, with the RNA 6000 Nano and the small RNA Nano chips, respectively (Agilent Technologies, Palo Alto, CA). Only total RNA samples with RNA Integrity Number (RIN) values ≥6 and with miRNA <20% were used for microarray analysis.

### MiRNA and gene expression profiling

MiRNA expression profiles were carried out in irradiated (0.2, 2Gy) vs. non-irradiated PBL, incubated for 4 and 24 h in 1 g and MMG. Analyses were performed by using the “Human miRNA Microarray kit (V2)” (Agilent Technologies), that allows the detection of 723 known human (miRBase v.10.1) and 76 human viral miRNAs. Total RNA (200 ng) was labeled with pCp Cy3, according to the Agilent protocol and unincorporated dyes were removed with MicroBioSpin6 columns (BioRad) [55]. Probes were hybridized at 55°C for 22 hours using the Agilent's Hybridization Oven that is suited for bubble-mixing and microarray hybridization processes. Then, the slides were washed by Agilent Gene expression wash buffer 1 and 2 and scanned using an Agilent microarray scanner (model G2565CA) at 100% and 5% sensitivity settings. Agilent Feature Extraction software version 10.5.1.1 was used for image analysis.

Gene expression profiling was carried out in 2Gy-irradiated vs. non-irradiated PBL, incubated for 24 h in 1 g or MMG, previously analyzed for miRNA profiling. We used the “Whole Human Genome Oligo Microarray” (Agilent), consisting of ∼41.000 (60-mer) oligonucleotide probes, which span conserved exons across the transcripts of the targeted full-length genes. 800 ng of total RNA were labeled with “Agilent One-Color Microarray-Based Gene Expression protocol” according to the manufacturer's instructions. 1.65 µg of labeled cRNA were used to prepare the hybridization samples and the hybridization was carried out at 65°C for 17 hours in a hybridization oven rotator (Agilent). The arrays were washed by Agilent Gene expression wash buffers and Stabilization and Drying Solution as suggest by the supplier. Slides were scanned on an Agilent microarray scanner (model G2565CA) and Agilent Feature Extraction software version 10.5.1.1 was used for image analysis. Raw data are available on the Gene Expression Omnibus (GEO) website (http://www.ncbi.nlm.nih.gov/geo/) using accession number GSE20120 for miRNA expression profiling (72 experiments) and accession number GSE20173 for mRNA expression profiling (20 experiments).

### Statistical analysis of miRNA and gene expression data

Inter-array normalization of expression levels was performed with cyclic Lowess for miRNA experiments and with quantile for gene expression profiling [56] to correct possible experimental distortions. Normalization function was applied to expression data of all experiments and then values of spot replicates within arrays were averaged. Furthermore, Feature Extraction Software provides spot quality measures in order to evaluate the goodness and the reliability of hybridization. In particular flag “glsFound” (set to 1 if the spot has an intensity value significantly different from the local background, 0 otherwise) was used to filter out unreliable probes: flag equal to 0 will be noted as “not available (NA)”. So, in order to make more robust and unbiased statistical analysis, probes with a high proportion of “NA” values were removed from the dataset. We decided to use the 40% of NA as threshold in the filtering process obtaining a total of 270 available human miRNAs. Principal component analysis, cluster analysis and profile similarity searching were performed with tMev that is part of the TM4 Microarray Software Suite [57]. The identification of differentially expressed genes and miRNAs was performed with one and two class Significance Analysis of Microarray (SAM) program [58] with default settings. The expression level of each miRNA and mRNA was calculated as the log 2 (irradiated/non-irradiated) PBL of the same donor.

### Identification of miRNA target genes and anti-correlation analysis of miRNA and mRNA expression data

To predict miRNA targets we have performed a computational analyses using PITA algorithm based on thermodynamic stability of the RNA-RNA duplex, considering free energy minimization [59]. PITA algorithm was applied over up-to-date version 38 of RefSeq transcript sequences and it used miRNAs sequences downloaded from mirBase version 14. To identify the most likely targets, we have integrated mRNA and miRNA expression data, obtained on the same biological samples, using MAGIA web tool [60]. We used a non-parametric index (Spearman correlation coefficient), the most indicated statistical coefficient for a small number of measures, to estimate the degree of anti-correlation (e.g. up-regulated miRNA and corresponding down-regulated mRNA target) between any putative pairs of miRNA and mRNA [61], [62] and we have selected as functional only those anti-correlated less than −0.775. To identify biological processes most involved in the biological phenomena under study we have performed a Gene Ontology (GO) analysis, using DAVID tool [63], on significant anti-correlated target genes identifying biological pathways significantly enriched (*P*<0.05). Pathway enrichment analysis was carried out by KEGG web tool [63] whereas miRNA-mRNA anti-correlations were visualized by Cytoscape software package [64].

### Validation of miRNA and mRNA expression levels with qRT-PCR

The data of miRNA expression analysis were validated by using the TaqMan® MicroRNA Assay kit (Applied Biosystems, Foster City, CA), that incorporate a target-specific stem-loop reverse transcription primer to provide specificity for the mature miRNA target. In brief, each RT reaction (15 µl) contained 10 ng of total purified RNA, 5× stem-loop RT primer, 1× RT buffer, 0.25 mM each of dNTPs, 50 U MultiScribe™ reverse transcriptase and 3.8 U RNase inhibitor. The reactions were incubated in a Mastercycler EP gradient S (Eppendorf) in 0.2 ml PCR tubes for 30 min at 16°C, 30 min at 42°C, followed by 5 min at 85°C, and then held at 4°C. The resulting cDNA was quantitatively amplified in 40 cycles on an ABI 7500 Real-Time PCR System, using TaqMan Universal PCR Master Mix and Taqman MicroRNA Assays for miR-34a, miR-424*, miR-181a-2*, miR-144, miR-598, miR-27a, and for U48 small nuclear (RNU48) as endogenous control.

For mRNA detection, 1 µg of total RNA was retrotranscribed with ImProm-II Reverse Transcription System (Promega). qRT-PCR was performed with the GoTaq qPCR Master Mix (Promega) and gene-specific primers for ATM, BAX, FANCF, STAT5A, TNFRSF10B genes and for GADPH as reference. qRT-PCR reactions were always performed in quadruplicates, in PBL samples from 4–6 donors. The relative expression levels of miRNAs and mRNAs between samples were calculated using the comparative delta *C*T (threshold cycle number) method (2-^ΔΔCT^) implemented in the 7500 Real Time PCR System software [65].

### Luciferase reporter assays

Luciferase reporter vectors containing the 3′-UTR of miR-27a, miR-144 and miR-424* target genes ATM, FANCF, STAT5A, TNFRSF10B, BAX, were generated following PCR amplification from human cDNA and cloned into the pmirGLO Dual-Luciferase miRNA Target Expression Vector (Promega, Madison, WI), immediately downstream from the stop codon of the luciferase gene. The sequence of each insert was confirmed by sequencing. MiR-27a-sensor, miR-144-sensor and miR-424*-sensor, were obtained by annealing, purifying and cloning short oligonucleotides containing the perfect miRNA binding site into the *Sac*I and *Xba*I sites of the pmirGLO vector. A549 cells were plated in 24-well plates (14×10^5^ cells/well) and 24 h later co-transfected with 50 ng of the pmirGLO dual-luciferase constructs, containing the indicated 3′UTRs of target genes, and with 32 nM pre-miR™ miRNA Precursor Molecules-Negative Control or pre-miR™ miRNA Precursor hsa-miR-27a (PM10939), hsa-miR-424*(PM12641), and hsa-miR-144 (PM11051) (all from Ambion, Austin, TX), using Lipofectamine2000 (Invitrogen Life Technologies). Lysates were collected 24 h after transfection and Firefly and Renilla Luciferase activities were consecutively measured by using Dual-Luciferase Reporter Assay (Promega) according to manufacturer's instructions. Relative luciferase activity was calculated by normalizing the ratio of Firefly/Renilla luciferase to that of negative control-transfected cells. Transfections were performed in triplicate and repeated 3–4 times.

## Results

### Radio-responsive miRNAs in static condition (1 g)

**Figure 2:**
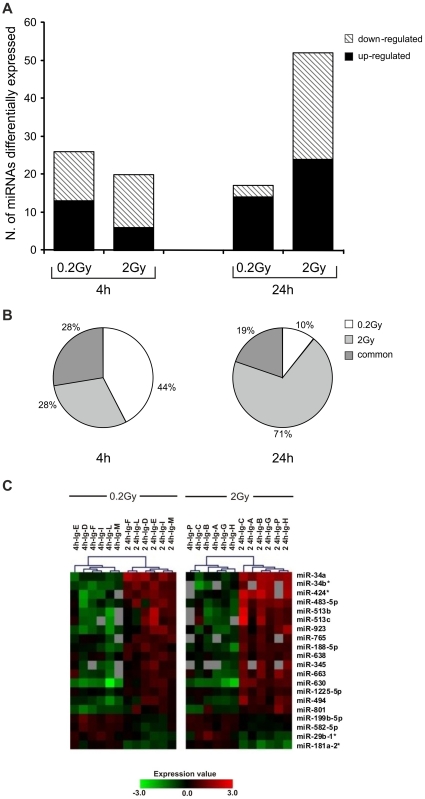
Differentially expressed miRNAs in irradiated PBL incubated in 1 g condition. (A) Number of radio-responsive miRNAs at 4 and 24 h after irradiation with 0.2 and 2Gy of γ-rays. (B) Percentage of dose-responsive miRNAs at the same time points. The expression level of each radio-responsive miRNA is the mean of expression values from six different donors/dose determined by the log2 (irradiated/non-irradiated) PBL. (C) Dendrogram showing radio-responsive miRNAs common to 0.2 and 2Gy of γ-rays, whose expression changed between 4 and 24 h after irradiation. Six different donors were analyzed for each dose, as indicated by the capital letters. The range of expression value is from -3.0 (green, down-regulation) to 3.0 (red, up-regulation). Grey boxes correspond to not available (N/A) fluorescent signal from the microarray platform.

Human PBL were irradiated *in vitro* with γ-rays (0.2Gy; 2Gy) and incubated in normal gravity (1 g) and in MMG during the post-irradiation time. Twelve total donors were analyzed, six for each dose, by performing independent experiments, in which irradiated and non-irradiated PBL of the same donor were incubated in 1 g and in MMG for 4 and 24 h. MicroRNA expression profiling was performed on total RNA extracted at the end of incubation times (Figure 1), by comparing the expression profile of irradiated vs. non-irradiated PBL of the same donor. Data obtained from PBL incubated in 1 g allowed to identify 26 (0.2Gy) and 20 (2Gy) radio-responsive miRNAs at 4 h after irradiation; miRNAs differentially expressed at 24 h after irradiation were 17 (0.2Gy) and 52 (2Gy), (Figure 2A and Table 1). Raw data and means of miRNA expression values are available on Table S1. Besides a fraction of radio-responsive miRNAs common between 0.2 and 2Gy (28% and 19% at 4 and 24 h after IR, respectively), most of miRNA species was activated in a dose-related manner (Figure 2B). Our results showed that, early after irradiation, both doses induced consistent changes in miRNA expression, whereas late after irradiation, the effect of the higher dose was predominant. Furthermore, most of radio-responsive miRNAs showed a time-related expression pattern, with a substantial down-regulation at 4 h and up-regulation at 24 h after IR (Figure 2C).

**Table 1:** Differentially expressed miRNAs in γ-irradiated versus non-irradiated human PBL.

4 h after irradiation			24 h after irradiation		
miRNA name	0.2Gy	2Gy	miRNA name	0.2Gy	2Gy
hsa-miR-21*	−0.84	−0.72	hsa-miR-1225-5p	0.44	0.60
hsa-miR-34b*	−0.89	−0.85	hsa-miR-135a*	0.48	0.67
hsa-miR-210	0.53	0.46	hsa-miR-152	−0.43	−0.41
hsa-miR-630	−1.75	−1.41	hsa-miR-181a-2*	−0.57	−0.99
hsa-miR-886-3p	−0.59	−0.91	hsa-miR-188-5p [46]	0.60	0.73
hsa-miR-199b-5p	0.58	0.39	hsa-miR-34a [44], [48], [52], [70], [71]	1.53	1.76
hsa-miR-582-5p	0.48	0.39	hsa-miR-34b* [68]	1.02	1.66
hsa-miR-378	−0.71	−0.46	hsa-miR-424*	1.00	2.08
hsa-miR-513b	−0.96	−0.65	hsa-miR-638 [49], [70]	0.57	0.59
hsa-miR-923	−0.99	−0.53	hsa-miR-663 [46], [49], [68], [70]	0.46	0.99
hsa-let-7e [45], [47], [49]	−0.31		hsa-miR-765	0.63	0.71
hsa-miR-16 [52]	0.30		hsa-miR-1226*	0.76	
hsa-miR-23a*	0.17		hsa-miR-150*	0.38	
hsa-miR-34a	−0.64		hsa-miR-202	0.43	
hsa-miR-145	0.75		hsa-miR-601	0.78	
hsa-miR-181b	0.30		hsa-miR-760	0.42	
hsa-miR-196b	0.55		hsa-miR-886-3p	−0.60	
hsa-miR-202	0.49		hsa-miR-100 [72]		−0.50
hsa-miR-221 [52], [69]	0.51		hsa-miR-101*		−0.37
hsa-miR-339-3p	−0.34		hsa-miR-10a		−0.57
hsa-miR-345	−0.62		hsa-miR-141		−0.38
hsa-miR-425*	0.43		hsa-miR-151-3p		−0.32
hsa-miR-450a	0.52		hsa-miR-16-2*		−0.66
hsa-miR-494	−0.76		hsa-miR-17 [69], [73]		−0.22
hsa-miR-629*	0.71		hsa-miR-181a [44], [69]		−0.31
hsa-miR-801	−0.85		hsa-miR-18b [52]		−0.35
hsa-miR-223		0.47	hsa-miR-196a		−0.91
hsa-miR-301a		0.45	hsa-miR-196b [70]		−0.51
hsa-miR-513a-5p		−1.19	hsa-miR-19b		−0.25
hsa-miR-940		0.53	hsa-miR-200b [70]		−0.24
hsa-miR-768-5p		−0.38	hsa-miR-210 [44], [53]		−0.31
hsa-miR-146a		−0.41	hsa-miR-221*		−0.36
hsa-miR-575		−0.47	hsa-miR-29b-1*		−0.47
hsa-miR-378*		−0.47	hsa-miR-30d [69]		−0.18
hsa-miR-188-5p		−0.59	hsa-miR-30e*		−0.24
hsa-miR-126* [69]		−0.60	hsa-miR-330-3p		−0.34
			hsa-miR-335		−0.35
			hsa-miR-345 [44], [53], [71]		1.16
			hsa-miR-363		0.99
			hsa-miR-371-5p		0.55
			hsa-miR-421		0.50
			hsa-miR-483-5p		1.33
			hsa-miR-494		0.87
			hsa-miR-505*		−0.40
			hsa-miR-513a-5p		1.06
			hsa-miR-513b		1.19
			hsa-miR-513c		1.22
			hsa-miR-551b		−0.40
			hsa-miR-574-5p		0.97
			hsa-miR-630 [68], [73]		0.96
			hsa-miR-769-5p		−0.34
			hsa-miR-801		0.66
			hsa-miR-873		−0.64
			hsa-miR-877*		0.72
			hsa-miR-923		0.89
			hsa-miR-940		0.49
			hsa-miR-95		−0.44
			hsa-miR-99a		−0.64

Irradiated and non-irradiated PBL of the same donors were incubated in static gravity (1 g) for 4 and 24 h, and miRNA expression profile was analyzed at the end of each incubation time. The expression value of each radio-responsive miRNA is the mean of expression levels calculated as the log2 (irradiated/non-irradiated) PBL from six donors/dose (see Table S1). References of miRNAs differentially expressed in other cell types following the exposure to different stressors are given in parentheses.

### Radio-responsive miRNAs in modeled microgravity (MMG)

**Figure 3:**
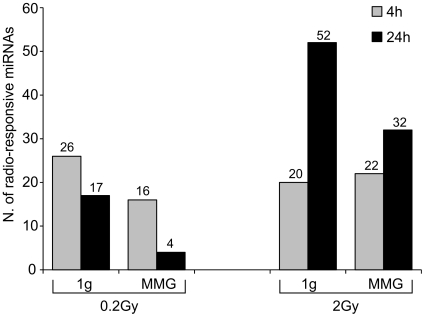
Effect of modeled microgravity on miRNA expression of irradiated PBL. Number of differentially expressed miRNAs in PBL irradiated with 0.2 and 2Gy and incubated for 4 and 24 h in MMG and in 1 g condition.

To check whether the expression of radio-responsive miRNAs was altered under modeled microgravity condition, we characterized the miRNA profile of PBL irradiated with 0.2 and 2Gy and incubated in MMG. After 4 h of incubation in MMG we identified 16 miRNAs responsive to 0.2Gy (vs. 26 in 1 g) and 22 miRNAs responsive to 2Gy (vs. 20 in 1 g). After 24 h of incubation in MMG we identified only 4 miRNAs responsive to 0.2Gy (vs. 17 in 1 g) and 32 miRNAs responsive to 2Gy (vs. 52 in 1 g) (Figure 3 and Table S2). The dose- and time-effect of IR on miRNA profile persisted in MMG, in spite of the decreased radio-responsiveness. In Figure S1 we report miRNAs which changed their expression level in a time-related pattern, showing the down-regulation at 4 h and the up-regulation at 24 h after irradiation, in common with 1 g.

**Figure 4:**
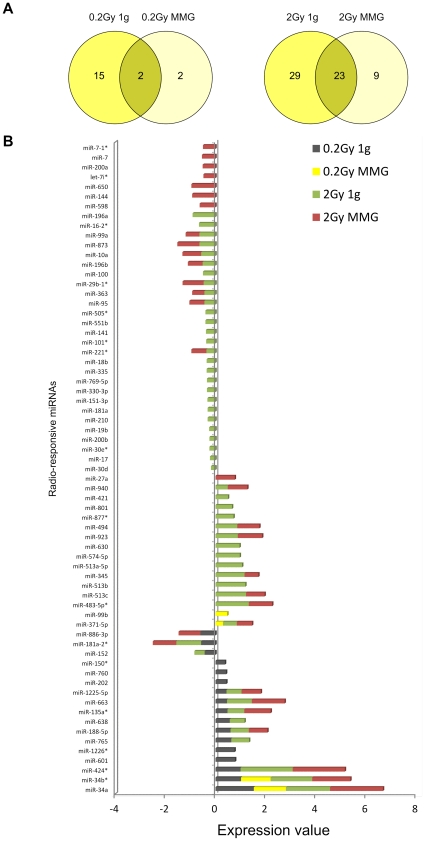
miRNA changes in human irradiated PBL incubated 24 h in 1 g and in MMG. A) The Venn Diagram shows the number of common and exclusively expressed miRNAs as a consequence of only radiation (0.2Gy 1 g, 2Gy 1 g) and radiation with microgravity (0.2Gy MMG, 2Gy MMG). B) miRNAs differentially expressed in PBL incubated 24 h in 1 g or MMG after irradiation with 0.2 and 2Gy. The expression level is given as log 2 (irradiated/non-irradiated) PBL incubated in the indicated gravity condition.

The Venn diagram in Figure 4A shows the number of shared and exclusively modulated miRNAs as a consequence of radiation alone (i.e. in 1 g) and radiation associated with MMG. Radio-responsive miRNAs specific to 1 g condition were 15 (0.2Gy) and 29 (2Gy), whereas those specific to MMG condition were 2 in 0.2Gy PBL and 9 in 2Gy PBL. Except for miR-371-5p and miR-886-3p, which were differently altered in 1 g at 24 h after irradiation (Table 1), miR-99b, let-7i*, miR-144, miR-200a, miR-27a, miR-598, miR-650, miR-7, miR-7-1* were activated by the combined exposure to IR and MMG (Figure 4B).

### Target prediction and integration analysis of miRNA and mRNA expression profiles

**Figure 5:**
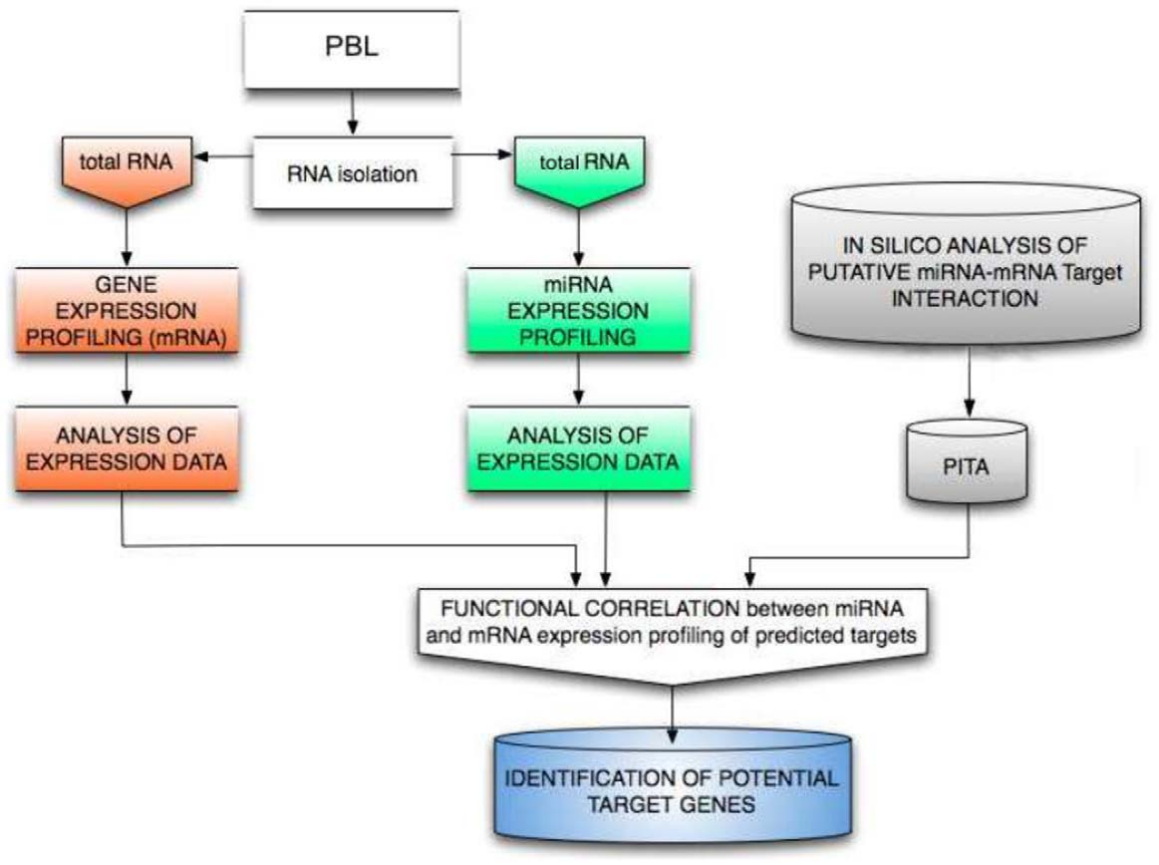
The procedure of computational prediction of miRNA targets. miRNA and gene expression profiles were defined in PBL irradiated with 2Gy versus non-irradiated PBL of the same donors, at the end of 24 h-incubation in 1 g and in MMG. Target prediction was first performed by PITA algorithm, then expression data were integrated to improve the detection of functional correlation between miRNA and mRNA expression profiling.

**Figure 6:**
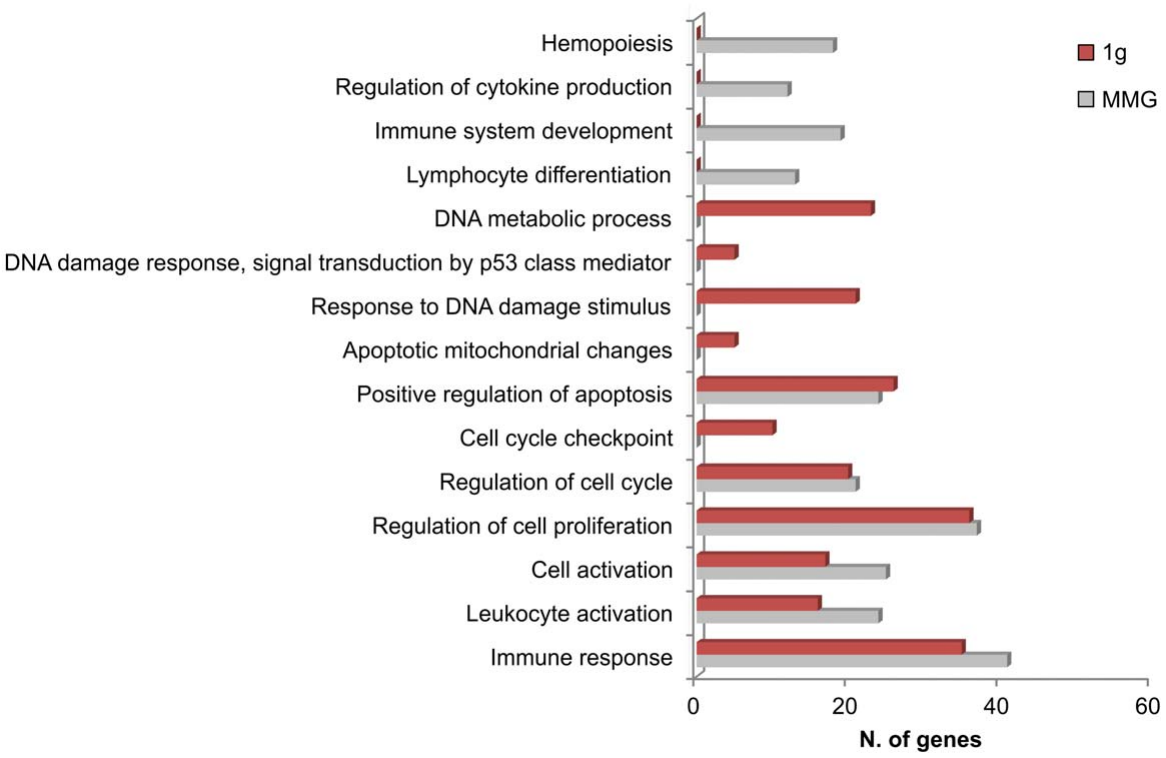
Gene Ontology (GO) analysis of anti-correlated target genes in 2Gy-irradiated PBL incubated 24 h in 1 g or MMG. Biological processes found enriched from GO analysis are shown. For each functional category is shown the number of genes differentially expressed in the two gravity condition.

To predict the target genes of differentially expressed miRNAs we first performed a computational analyses using PITA algorithm available on line [46], [59]. However, all available softwares for target prediction are characterized by a large fraction of false positive, thus, the integration of target predictions with miRNA and gene target expression profiles has been proposed to refine functional miRNA-mRNA relationships. For this purpose, we defined the gene expression signature on the same PBL samples used to assess miRNA expression profile, and then we compared miRNA and mRNA expression levels (Figure 5). Gene expression profiles were carried out only in 2Gy PBL incubated for 24 h in 1 g or in MMG, since for this dose and this time point, several IR-responsive genes involved in DDR pathway were previously identified in human quiescent PBL [66] and in human peripheral blood [67]. In addition, our previous results demonstrated that cell survival, double-strand break (DSB) repair, mutant frequency and apoptosis, were significantly altered when 2Gy PBL were incubated for 24 h in MMG [33], [54]. We did not perform gene expression profiling in 0.2Gy PBL incubated 24 h in 1 g or in MMG, since the number of miRNAs differentially expressed in MMG was too small (n = 4, Figure 3) to obtain informative data from the anti-correlation analysis. The 52 (in 1 g) and 32 (in MMG) differentially expressed miRNAs in 2Gy PBL resulted anti-correlated to a total of 379 and 391 transcripts, respectively. These transcripts were then classified according to DAVID [63], to determine which Gene Ontology (GO) terms were significantly enriched in our set of genes, in relation to the different gravity conditions. Biological categories common to 1 g and MMG were those of “Immune response”, “Leukocyte activation”, “Cell activation”, “Regulation of cell proliferation”, “Regulation of cell cycle”, “Positive regulation of apoptosis” (Figure 6). Biological categories of “Response to DNA damage stimulus”, “DNA damage response, signal transduction by p53 class mediator”, “Apoptotic mitochondrial changes” and “DNA metabolic process” were enriched only when PBL were incubated in 1 g. In MMG, instead were enriched categories of “Hemopoiesis”, “Regulation of cytokine production”, “Immune system development” and “Lymphocyte differentiation”, all characterized by a general gene down-regulation of gene expression (not-shown). By pathway enrichment analysis using the KEGG web tool [63] we found that the p53-pathway was enriched in both gravity conditions, but with some differences between 1 g and MMG (Figure S2). Table 2 provides the identity and associated functions of each altered gene belonging to p53 pathway, together with additional DDR genes found deregulated from miRNA-mRNA integrated analyses. Genes in italic are included in the GO category “Response to DNA damage stimulus”, which was enriched in 1 g but not in MMG.

**Table 2:** Significantly anti-correlated genes involved in the DNA-damage response to IR of human PBL.

Gene symbol	Gene name	Function	2Gy 1 g	2Gy MMG
			F.C.	F.C.
**ATM**	Ataxia Telangiectasia Mutated	DNA damage signal transduction; cell cycle checkpoint	- -	−1.42
***BAX***	BCL2-associated X protein	Apoptosis	3.40	3.22
***BBC3***	BCL2-binding component 3 (PUMA)	Apoptosis	5.94	- -
**CCND2**	Cyclin D2	Cell cycle progression	−1.81	−1.59
**CCNG1**	Cyclin G1	Cell cycle progression/arrest	2.83	- -
***CDC2***	Cell division cycle 2 (CDK1)	Cell cycle progression	−1.94	−2.48
***CDKN1A***	Cyclin-dependent kinase inhibitor 1A (p21)	Cell cycle arrest	3.89	- -
**CDKN2A**	Cyclin-dependent kinase inhibitor 2A (p14ARF)	DNA damage sensor; cell cycle arrest; apoptosis	−1.61	- -
***DDB2***	Damage-specific DNA binding protein 2 (p48)	DNA repair	4.89	- -
**EI24**	Tumor protein p53 inducible protein 8 (PIGs)	Apoptosis p53-dependent	1.46	1.40
***GADD45A***	Growth arrest and DNA-damage-inducible, alpha	Growth arrest; DNA repair; apoptosis	6.23	- -
**GADD45B**	Growth arrest and DNA-damage-inducible, beta	Growth arrest; apoptosis	- -	−2.54
**MDM2**	Mdm2 p53 binding protein homolog	Inactivation of tumour protein p53	3.75	2.86
**PPM1D**	Protein phosphatase 1D magnesium-dependent (Wild type p53-induced phosphatase,Wip1)	DNA damage sensor (phosphorylates H2AX)	1.49	- -
**RRM2B**	p53-inducible ribonucleotide reductase small subunit (p53R2)	DNA repair	- -	1.76
***SESN1***	Sestrin 1 (Sestrins)	Cell cycle arrest	2.56	- -
***THBS1***	Thrombospondin 1 (TSP1)	Cell growth	2.44	- -
**TNFRSF10B**	Tumor necrosis factor receptor superfamily, member 10b (DR5)	Apoptosis	2.60	3.65
**TP53**	Tumor protein p53	Cell cycle arrest; DNA repair; apoptosis	- -	−1.73
**TP53I3**	Tumor protein p53 inducible protein 3 (PIGs)	Apoptosis p53-dependent	1.72	- -
***ZMAT3***	Zinc finger, matrin type 3 (PAG608)	Cell growth; apoptosis p53-dependent	4.60	- -
*AEN*	Apoptosis Enhancing Nuclease	Apoptosis	5.42	4.44
BIRC5	Baculoviral IAP repeat-containing 5	Apoptosis	−2.90	−3.43
CDKN1C	Cyclin-dependent kinase inhibitor 1C (p57)	Cell cycle	2.11	4.22
*FANCA*	Fanconi anemia, complementation group A	DNA repair	−1.56	- -
FANCF	Fanconi anemia, complementation group F	DNA repair	- -	−1.47
FDXR	Ferrodoxin reductase	DNA damage, apoptosis	10.20	- -
IER5	Immediate early response 5	Apoptosis	1.86	- -
*LIG1*	Ligase I	DNA Repair	0.79	- -
MYC	v-myc myelocytomatosis viral oncogene homolog	Cell growth; apoptosis	−2.48	−2.88
*PCNA*	Proliferating cell nuclear antigen	DNA repair	2.41	2.60
*PHLDA3*	Pleckstrin homology-like domain, family A, member 3	Apoptosis	7.57	9.98
STAT5A	Signal transducer and activator of transcription 5A	Apoptosis	- -	−1.52
TNFRSF10D	Tumor necrosis factor receptor superfamily, member 10d	Apoptosis	1.75	- -
*TRIAP1*	TP53 regulated inhibitor of apoptosis 1	Apoptosis	2.34	2.88
*XPC*	Xeroderma pigmentosum, complementation group C	DNA repair	2.36	2.08

Significantly anti-correlated genes of DDR pathway in 2Gy PBL incubated for 24 h in 1 g or in MMG. Fold change (F.C.) is the mean of the expression values obtained from the transformed log2 (irradiated/non-irradiated) PBL (see Table S5 and Table S6). Genes in bold belong to p53-pathway; genes in italic belong to the GO category “Response to DNA damage stimulus”.

**Figure 7:**
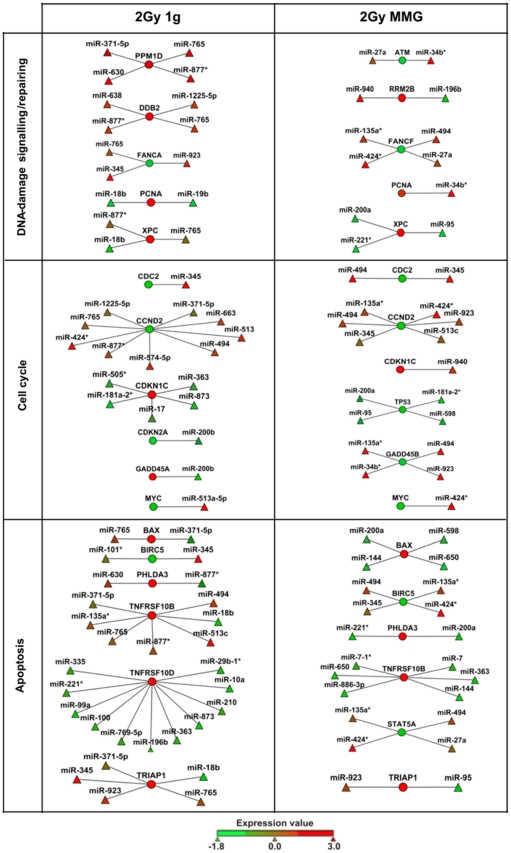
Visualization of functional miRNA-mRNA anti-correlation in the main pathways of DNA-damage response. Analyses were carried out in 2Gy-irradiated PBL incubated for 24 hours in gravity 1 g or in MMG. Circles represent transcripts and triangles miRNAs; the expression levels of each features are represented as color scale. The lists of significantly miRNA-mRNA anti-correlations are reported in Table S3 and Table S4.

By using Cytoscape [64] we were able to visualize miRNA-mRNA interactions of DDR pathway in the two gravity conditions (Figure 7). Our results show that the same transcript deregulated in both 1 g and MMG (i.e. PCNA, XPC, CDKN1C, BAX, BIRC5, PHLDA3, TNFRSF10B, TRIAP1) was, in most cases, anti-correlated to different miRNA species, according to the condition of gravity.

**Figure 8:**
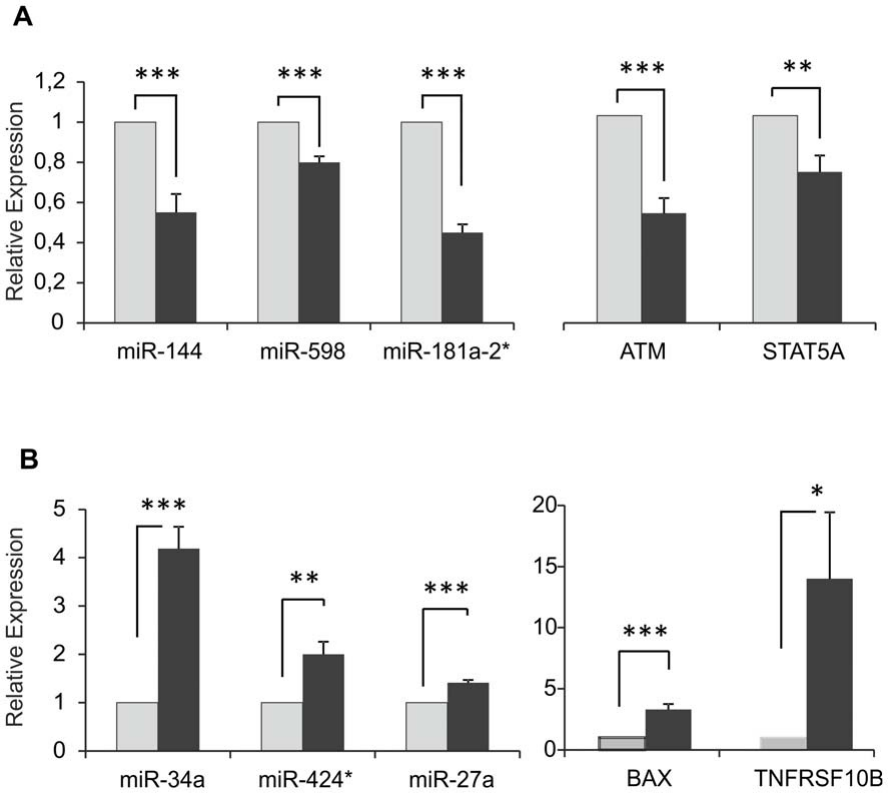
Microarray data validation by quantitative real-time PCR (qRT-PCR). Validation of microarray data by qRT-PCR in irradiated (2Gy) vs. non-irradiated PBL. A) Down-regulated miRNAs (miR-144, miR-598, miR-181a-2*) and mRNAs (ATM, STAT5A); B) Up-regulated miRNAs (miR-34a, miR-424*, miR-27a) and mRNAs (BAX, TNFRSF10B). Values (fold change, dark grey bars) are means ± S.E. of independent experiments performed in quadruplicate on PBL samples from 4–6 different donors. The value “1” of control PBL (light grey bars) is arbitrarly given when no change is observed (***P<0.001; **P<0.01, *P<0.05, *t*-test).

The microarray data from miRNA and mRNA expression profiling were validated by real time-qPCR experiments for six miRNAs and four mRNAs, whose expression level was significantly altered in response to radiation (Figure 8). MiR-27a, miR-144, miR-598, together with ATM and STAT5A transcripts, were down-regulated in 2Gy MMG samples. MiR-34a, miR-424*, miR-181a-2*, together with BAX and TNFRSF10B transcripts, were deregulated in 2Gy PBL incubated in both gravity conditions.

### Functional correlation between miRNAs and some of their potential target mRNAs

**Figure 9:**
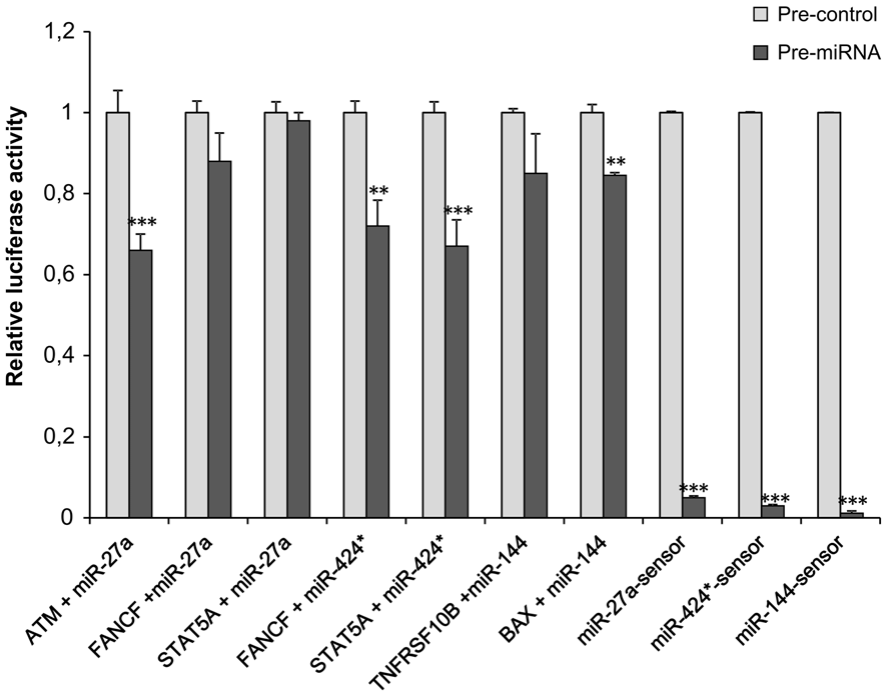
Luciferase reporter assay. Transient transfection analysis for luciferase expression in A549 cells co-transfected with pre-miRNA precursors (pre-miRNAs) miR-27a, miR-144, miR-424*, or pre-miRNA precursor-Negative Control (pre-control), and reporter constructs containing the 3′UTR of the indicated target genes or synthetic sequence including the perfect miR-27a, miR-144 and miR-424* binding site (sensors). Results are shown as mean ± S.D. of Firefly luciferase activity relative to controls, normalized on Renilla luciferase activity, from 3–4 independent experiments (***P<0.001, **P<0.01, *t*-test).

The modulation of miRNAs involved in the DNA-damage response to radiation showed some differences between samples incubated in normal gravity and microgravity. To validate some of the predicted miRNA-mRNA anti-correlations identified in 2Gy-PBL incubated in 1 g and MMG, we selected three miRNAs (miR-27a, miR-424*, miR-144) and at least 2 of their potential targets, for functional testing *in vitro* with the luciferase reporter assay. While miR-424* was up-regulated in response to radiation in both gravity conditions, miR-27a and miR-144 were respectively up-regulated and down-regulated, in 2Gy-PBL incubated in MMG. A549 cells were co-transfected with the synthetic miRNA precursors (pre-miRNA) of interest or miRNA precursor-negative control (pre-control), and expression vectors containing the 3′UTR of the target gene, cloned downstream of the luciferase gene. The results show that pre-miR-27a reduced significantly the luciferase activity from constructs containing the ATM 3′UTR, but not the FANCF and STAT5A 3′UTR. Pre-miR-424* reduced significantly the luciferase activity of both FANCF and STAT5A 3′UTR containing vectors, whereas pre-miR-144 reduced significantly that from constructs containing the BAX 3′UTR (Figure 9). As positive controls were used miR-27a-sensor, miR-144-sensor, and mR-424*-sensor constructs, containing the perfect binding site for each miRNA.

## Discussion

With the present study, carried out on human PBL, we evaluated whether the expression of miRNAs involved in the DNA-damage response to γ-radiation was affected by modeled microgravity incubation during the repair time, enhancing the biological effects of ionizing radiation. In ground gravity the exposure to γ-radiation alters miRNA expression profile as a function of the dose and the time after IR. These results are in accordance with data from normal human fibroblasts [46], [53] and lymphoblastic IM9 cells [68], but not with those from lymphoblastoid TK6 cells [44], and from human endothelial cells [69], strengthening the existence of cell-specific miRNA radio-response. Several of the radio-responsive miRNAs here identified have been found deregulated in other cells types exposed to different kind of stressors (Table 1), [44]–[49], [52], [53], [68]–[73].

Also modeled microgravity alters miRNA expression profile of irradiated PBL in a dose- and time-manner, but with a decreased radio-responsiveness compared with in 1 g. Indeed, 15 and 29 miRNAs were not responsive to 0.2 and 2Gy, respectively, when incubation occurred in MMG. Due to the decrease of radio-responsiveness of miRNA expression, we believe that the DNA-damage response to radiation might be affected by MMG incubation during the repair time. From our analyses we individuated common and distinct miRNAs differentially expressed in response to radiation alone (in 1 g) or associated with MMG. Eight miRNAs (let-7i*, miR-7, miR-7-1*, miR-27a, miR-144, miR-200a, miR-598, miR-650), were differentially expressed only when PBL were incubated in MMG. Probably, they not respond to the direct insult of γ-radiation, but are instead altered by the combined action of ionizing radiation and microgravity.

To improve the detection of functional miRNA-mRNA relationships, we analyzed the gene expression profiles on the same PBL samples used to assess miRNA expression levels. Then, we integrated the transcriptome and microRNome and we performed the anti-correlation analysis. Gene Ontology (GO) analysis was conducted on significant anti-correlated target genes to identify common and distinct biological categories significantly over-represented in our set of anti-correlated transcripts. Our results show that most genes were gravity-specific. While in PBL incubated in 1 g a great number of hits were achieved by the categories of DDR pathway, in PBL incubated in MMG these categories were not enriched. We instead found enriched processes involved in hemopoiesis, regulation of cytokine production, immune system development and lymphocyte differentiation.

To investigate on the differences of DDR pathway between 1 g and MMG conditions, we focused on p53-pathway, which is pivotal in eliciting a complex DDR in response to genotoxic and non-genotoxic stresses [74]–[77]. The ATM transcript resulted down-regulated in MMG and anti-correlated to miR-27a, as confirmed by functional validation with the luciferase reporter assay. Mir-27a, which is activated by the combined action of IR and MMG, is abnormally up-regulated in several types of cancers and has been identified to play an oncogenic role in the progression of cancers [78]–[81]. Moreover, miR-27a is a member of the miR-24 cluster, whose over-expression enhances TNF-a induced apoptosis in human embryonic kidney cells [82] and affects DSB repair in terminally differentiated blood cells by deregulating H2AX expression [83].

The results of anti-correlation analyses reported the down-regulation of p53 transcript (TP53) and alterations in the expression levels of several p53-related transcripts (BBC3, CCNG1, CDKN1A, CDKN2A, DDB2, GADD45A, PPM1D, RRM2B,SESN1,THBS1, TP53I3, ZMAT3), when 2Gy PBL were incubated in MMG. PPM1D transcript encodes for the p53-induced protein phosphatase 1 (WIP1), which modulates the expression of H2AX, the histone with a key role in DNA damage signaling and DSB repairing [84]. Since overexpression of WIP1 facilitates the clearance of IR-induced γ-H2AX foci after DNA repair is completed [85], the up-regulation of WIP1 transcript observed in 1 g but not in MMG, is consistent with our previous results, showing s slower disappearance of γ-H2AX foci in irradiated PBL incubated in MMG [33]. From our results the extrinsic pathway of apoptosis appeared more targeted in MMG than in 1 g. Indeed, miR-7, miR-7-1*, miR-144 and miR-650, which were anti-correlated to pro-apoptotic TNFRSF10B, were exclusively radio-responsive in MMG. The expression level of the gene, which encodes the receptor for the cytotoxic ligand TNFSF10/TRAIL, was 3.65-fold induced in MMG vs. 2.6-fold in 1 g. Also PHLDA3, which contributes to p53/TP53-dependent apoptosis by repressing AKT1 activity, showed a higher expression level in MMG. This gene was anti-correlated to miR-200a, itself activated by the associated action of IR and MMG. On the contrary, the intrinsic apoptotic pathway appeared more targeted in 1 g, with four up-regulated genes (BBC3, TP53I3, ZMAT3, FDXR). Notably, pro-apoptotic BAX was anti-correlated to four miRNAs (miR-144, miR-200a, miR-598,miR-650) deregulated in only 2Gy MMG PBL. The luciferase assay confirmed the functional interaction between miR-144 and BAX, suggesting that this miRNA may contribute to, or amplify, IR-induced apoptosis in MMG. Instead, the interaction between miR-144 and miR-27a with their putative target genes involved in apoptosis (respectively TNFRSF10B and STAT5A), diminished the luciferase activity, but not significantly. It should be noted that such transcripts are anti-correlated to 4–6 different miRNAs that can act together on individual mRNA to produce additive or synergistic effects [86], [87].

On the whole our results evidence that 1) many transcripts are targeted by multiple miRNAs; 2) the same transcript can be targeted by different miRNA species according to the gravity condition; 3) miRNAs responsive to IR in both gravities not always target the same transcript. The possibility that a single mRNA can be targeted by multiple miRNAs has been reported [88], [89] and recently demonstrated by Wu et al. [86]. The reason why the same mRNA could be anti-correlated to different miRNA species in relation to the gravity condition, is not clear. We hypothesize that the reduced number of radio-responsive miRNAs in MMG can operate an unscheduled regulation of transcripts. Moreover, the dysregulation of mRNAs caused by microgravity-mediated gene expression changes, could impact on miRNAs recruitment. This idea is consistent with the recent findings of Poliseno and co-authors [90] which demonstrated that mRNAs introduced into a cell can potentially perturb the interaction between miRNAs and their multiple targets, thus having a biological activity independent of the translation of the protein they encode. These authors propose that mRNAs could possess a regulatory role that relies on their ability to compete for miRNA binding, independently of their protein-coding function. Furthermore, it has been shown that miRNAs themselves are subjected to post-transcriptional regulation. Various proteins induce processing of specific pri-miRNAs into mature miRNAs by influencing the Drosha-dgcr8 complex, while other proteins block miRNAs maturation by binding to the pre-miRNA [91]–[93]. Thus, microgravity could affect post-transcriptional regulation of miRNAs. In addition, since miRNA biogenesis is globally induced upon DNA damage in an ATM-dependent manner and the loss of ATM abolishes miRNA induction after DNA damage [94], we suggest that ATM down-regulation in MMG could affect miRNA biogenesis in response to radio-induced DNA damage.

In conclusion, the miRNA-mRNA changes observed in 2Gy PBL incubated in MMG conditions may translate into alterations of DDR pathway, at the DNA level and/or in the processing of IR-induced DNA damage, that could affect cell survival, mutation rate and apoptosis induction.

## Supporting Information

Figure S1 **Radio-responsive miRNAs common to 1 g and MMG, changed as a function of the time after irradiation.** Heatmap of differentially expressed miRNAs at 4 and 24 h after irradiation with 0.2Gy (A) and 2Gy (B), common to 1 g- and MMG-incubated PBL. Range of expression value expressed as log2 (irradiated/non-irradiated) PBL is from −2.0 (green, down-regulation) to 2.0 (red, up-regulation).
(TIFF)

Figure S2 **p53 regulatory pathway.** Network diagram of KEGG p53 signaling pathway in human PBL incubated 24 h in 1 g (A) or in MMG (B) after irradiation with 2Gy. Over-expressed (red), under-expressed (dark green) and not significantly changed (light green) transcripts are shown. The identity and associated functions of each altered gene are given in Table 2 of the text.
(TIFF)

Table S1 **miRNAs differentially expressed in 1 g-incubated PBL.** This table lists differentially expressed miRNAs in human PBL at 4 and 24 h after irradiation with 0.2 and 2Gy and incubation in ground condition (1 g). The table includes miRNA ID and the expression value of each sample expressed as log2 (irradiated/non-irradiated) PBL.
(XLS)

Table S2 **miRNAs differentially expressed in MMG-incubated PBL.** This table lists differentially expressed miRNAs in human PBL at 4 and 24 h after irradiation with 0.2 and 2Gy and incubation in modeled microgravity (MMG). The table includes miRNA ID and the expression value of each sample expressed as log2 (irradiated/non-irradiated) PBL.
(XLS)

Table S3 **Functional anti-correlations in 1 g.** List of the most significant miRNA-mRNA relationships (Spearman index <−0.775) performed by MAGIA, in human PBL at 24 h after irradiation with 2Gy and incubation in 1 g. The table includes RefSeq ID, gene symbol, mRNA description, anti-correlated miRNA, Spearman correlation index, gene and miRNA expression levels of each putative anti-correlated pairs of miRNA and mRNA in the same sample.
(XLS)

Table S4 **Functional anti-correlations in MMG.** List of the most significant miRNA-mRNA relationships (Spearman index <−0.775) performed by MAGIA, in human PBL at 24 h after irradiation with 2Gy and incubation in modeled microgravity. The table includes RefSeq ID, gene symbol, mRNA description, anti-correlated miRNA, Spearman correlation index, gene and miRNA expression levels of each putative anti-correlated pairs of miRNA and mRNA in the same sample.
(XLS)
