# Supplementary figures and images for: Analysis of miRNA and mRNA Expression Profiles Highlights Alterations in Ionizing Radiation Response of Human Lymphocytes under Modeled Microgravity
Source: PLoS One. 2012 Feb 9;7(2):e31293. doi: 10.1371/journal.pone.0031293 (PMC3276573; doi:10.1371/journal.pone.0031293)

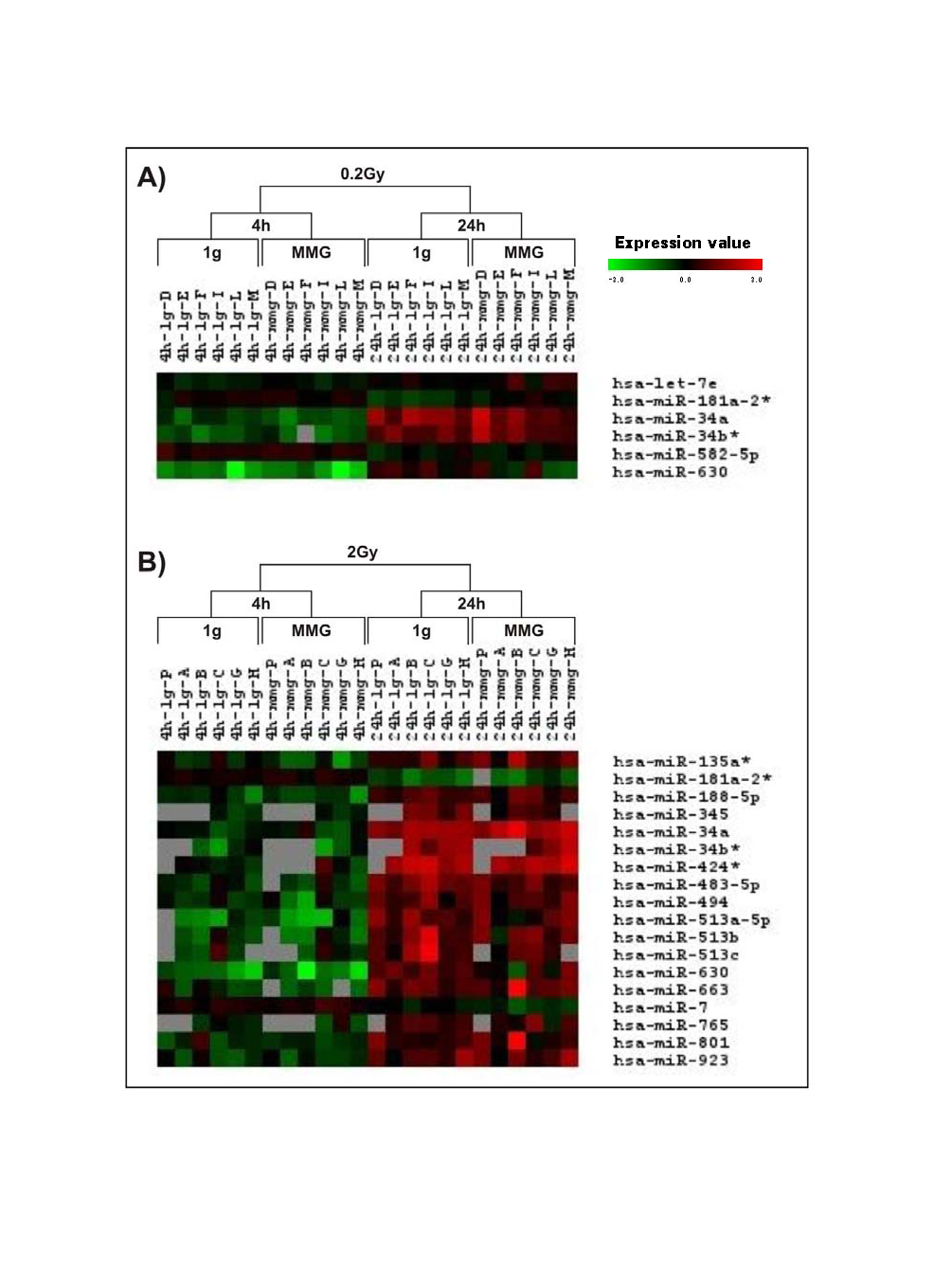

Supplement: Figure S1 — Radio-responsive miRNAs common to 1 g and MMG, changed as a function of the time after irradiation. Heatmap of differentially expressed miRNAs at 4 and 24 h after irradiation with 0.2Gy (A) and 2Gy (B), common to 1 g- and MMG-incubated PBL. Range of expression value expressed as log2 (irradiated/non-irradiated) PBL is from −2.0 (green, down-regulation) to 2.0 (red, up-regulation). (TIFF) [file pone.0031293.s001.tiff]

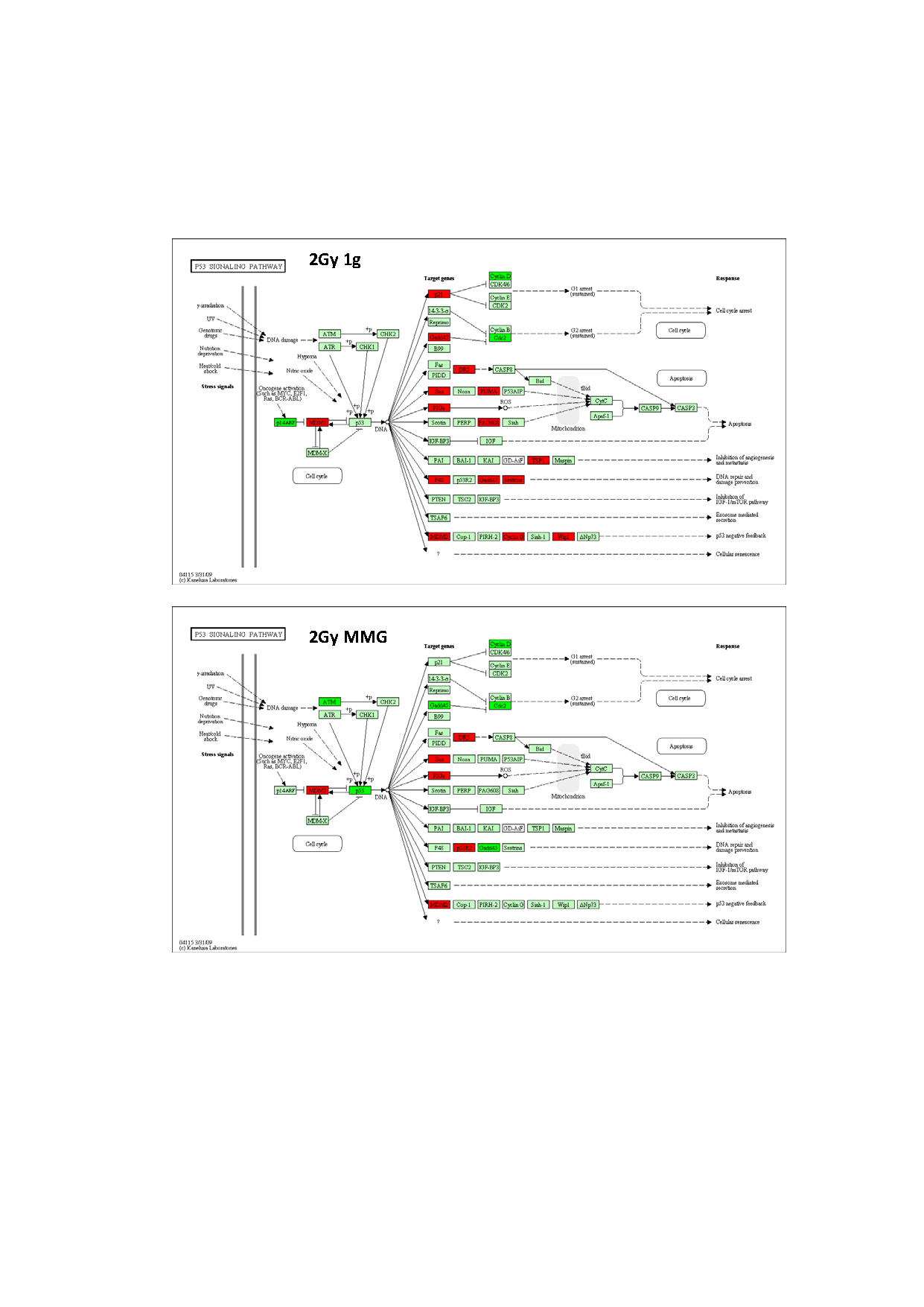

Supplement: Figure S2 — p53 regulatory pathway. Network diagram of KEGG p53 signaling pathway in human PBL incubated 24 h in 1 g (A) or in MMG (B) after irradiation with 2Gy. Over-expressed (red), under-expressed (dark green) and not significantly changed (light green) transcripts are shown. The identity and associated functions of each altered gene are given in Table 2 of the text. (TIFF) [file pone.0031293.s002.tiff]
